# Supplementary material for: Bayesian refinement of protein structures and ensembles against SAXS data using molecular dynamics
Source: PLoS Comput Biol. 2017 Oct 18;13(10):e1005800. doi: 10.1371/journal.pcbi.1005800 (PMC5662244; doi:10.1371/journal.pcbi.1005800)
Supplement: S1 Archive — (BZ2) [file pcbi.1005800.s011.tar.bz2 › gromacs_4.62_bayesian_saxs_refi/share/html/online/g_kinetics.html]

g\_kinetics


| g\_kinetics Main Table of Contents |

**VERSION 4.6  
Sat 19 Jan 2013**

---

### Description

g\_kinetics reads two .xvg files, each one containing data for N replicas.
The first file contains the temperature of each replica at each timestep,
and the second contains real values that can be interpreted as
an indicator for folding. If the value in the file is larger than
the cutoff it is taken to be unfolded and the other way around.

From these data an estimate of the forward and backward rate constants
for folding is made at a reference temperature. In addition,
a theoretical melting curve and free energy as a function of temperature
are printed in an .xvg file.

The user can give a max value to be regarded as intermediate
(-ucut), which, when given will trigger the use of an intermediate state
in the algorithm to be defined as those structures that have
cutoff < DATA < ucut. Structures with DATA values larger than ucut will
not be regarded as potential folders. In this case 8 parameters are optimized.

The average fraction foled is printed in an .xvg file together with the fit to it.
If an intermediate is used a further file will show the build of the intermediate and the fit to that process.

The program can also be used with continuous variables (by setting
-nodiscrete). In this case kinetics of other processes can be
studied. This is very much a work in progress and hence the manual
(this information) is lagging behind somewhat.

In order to compile this program you need access to the GNU
scientific library.

### Files

| option | filename | type | description |
| --- | --- | --- | --- |
| **-f** | temp.xvg | Input | xvgr/xmgr file |
| **-d** | data.xvg | Input | xvgr/xmgr file |
| **-d2** | data2.xvg | Input, Opt. | xvgr/xmgr file |
| **-o** | ft\_all.xvg | Output | xvgr/xmgr file |
| **-o2** | it\_all.xvg | Output, Opt. | xvgr/xmgr file |
| **-o3** | ft\_repl.xvg | Output, Opt. | xvgr/xmgr file |
| **-ee** | err\_est.xvg | Output, Opt. | xvgr/xmgr file |
| **-g** | remd.log | Output | Log file |
| **-m** | melt.xvg | Output | xvgr/xmgr file |

### Other options

| option | type | default | description |
| --- | --- | --- | --- |
| **-[no]h** | bool | no | Print help info and quit || **-[no]version** | bool | no | Print version info and quit || **-nice** | int | 19 | Set the nicelevel || **-tu** | enum | ps | Time unit: fs, ps, ns, us, ms or s || **-[no]w** | bool | no | View output .xvg, .xpm, .eps and .pdb files || **-xvg** | enum | xmgrace | xvg plot formatting: xmgrace, xmgr or none || **-[no]time** | bool | yes | Expect a time in the input || **-b** | real | 0 | First time to read from set || **-e** | real | 0 | Last time to read from set || **-bfit** | real | -1 | Time to start the fit from || **-efit** | real | -1 | Time to end the fit || **-T** | real | 298.15 | Reference temperature for computing rate constants || **-n** | int | 1 | Read data for this number of replicas. Only necessary when files are written in xmgrace format using @type and & as delimiters. || **-cut** | real | 0.2 | Cut-off (max) value for regarding a structure as folded || **-ucut** | real | 0 | Cut-off (max) value for regarding a structure as intermediate (if not folded) || **-euf** | real | 10 | Initial guess for energy of activation for folding (kJ/mol) || **-efu** | real | 30 | Initial guess for energy of activation for unfolding (kJ/mol) || **-ei** | real | 10 | Initial guess for energy of activation for intermediates (kJ/mol) || **-maxiter** | int | 100 | Max number of iterations || **-[no]back** | bool | yes | Take the back reaction into account || **-tol** | real | 0.001 | Absolute tolerance for convergence of the Nelder and Mead simplex algorithm || **-skip** | int | 0 | Skip points in the output .xvg file || **-[no]split** | bool | yes | Estimate error by splitting the number of replicas in two and refitting || **-[no]sum** | bool | yes | Average folding before computing χ^2 || **-[no]discrete** | bool | yes | Use a discrete folding criterion (F <-> U) or a continuous one || **-mult** | int | 1 | Factor to multiply the data with before discretization |

---

http://www.gromacs.org  
gromacs@gromacs.org
